# 

**Published:** 1871-12

**Authors:** 


VOL. XXV. No. 12.

### THE
### Dental Register.

EDITED BY

J. TAFT & G. WATT.

DECEMBER, 1871.

MONTHLY:
THREE DOLLARS PER ANNUM, IN ADVANCE.

CINCINNATI:
WRIGHTSON & CO., Printers, 167 WALNUT STREET.

*J. & WM. TAFT, Dentists, 117 West Fourth St.*

